# A Bridging Opportunities Work-frame to develop mobile applications for clinical decision making

**DOI:** 10.4155/fso.15.5

**Published:** 2015-11-01

**Authors:** Tibor van Rooij, Serena Rix, James B Moore, Sharon Marsh

**Affiliations:** 1 Faculty of Pharmacy & Pharmaceutical Sciences, 3142, Katz Group Centre for Pharmacy & Health Research, University of Alberta, Edmonton, Alberta, AB T6G 2E1, Canada; 2 Pharmacy Department, Grey Nuns Community Hospital, 1100 Youville Drive West NW, Edmonton, Alberta, AB T6L 5X8, Canada; 3 Department of Biology, Faculty of Science, 1–001 Centennial Centre for Interdisciplinary Sciences, University of Alberta, Edmonton, Alberta, AB T6G 2E9, Canada

**Keywords:** clinical decision support, clinical guidance, eHealth, mHealth, mobile application, model development, point-of-care

## Abstract

**Background:**

Mobile applications (apps) providing clinical decision support (CDS) may show the greatest promise when created by and for frontline clinicians. Our aim was to create a generic model enabling healthcare providers to direct the development of CDS apps.

**Methods:**

We combined Change Management with a three-tier information technology architecture to stimulate CDS app development.

**Results:**

A Bridging Opportunities Work-frame model was developed. A test case was used to successfully develop an app.

**Conclusion:**

Healthcare providers can re-use this globally applicable model to actively create and manage regional decision support applications to translate evidence-based medicine in the use of emerging medication or novel treatment regimens.

## Abstract (lay abstract)

Medical information needs to be structured in a way that it can be used in a time-constrained environment. This project looked at the process of creating mobile applications (apps), to help medical professionals rapidly apply new knowledge to better treat patients. We developed a novel system that allowed medical professionals to have a leading role in development. With the input from a pharmacist we created an app to deal with pharmacy and patient-related decisions surrounding newly available anticancer pills. Other medical professionals could also use this method to make apps to provide information with relevance to their medical decisions.

**Figure 1.**
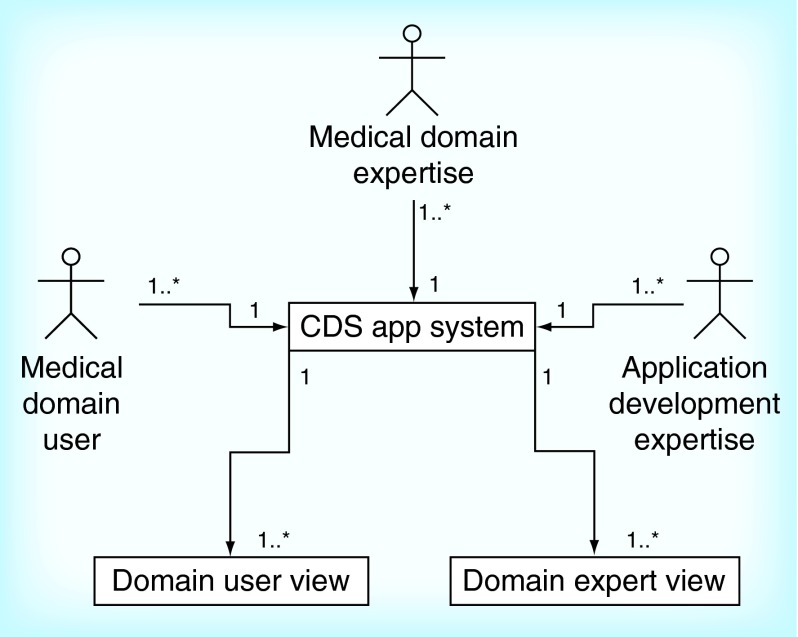
**A view of the stakeholders in a clinical decision support mobile application context when organized according to the Bridging Opportunities Work-frame model.** The medical domain expert is the main driver of the application development. The expert identifies the opportunity, in other words, the gap in the current workflow that would benefit from the introduction of CDS, and provides the curated knowledge that needs to be implemented into the CDS app system to help improve decision making. app: Mobile application; CDS: Clinical decision support.

**Figure 2.**
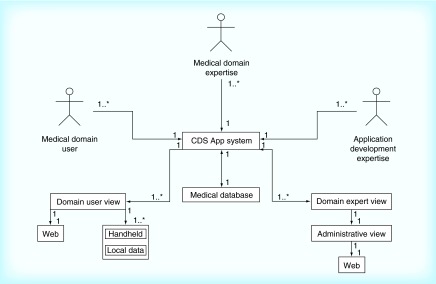
**Building on the outline shown in Figure 1, stakeholders’ actions, functional components and overall data flow are further defined.** CDS data are contained in a centralized transactional database (DB), which connects to both web views and to the mobile user counterpart through the CDS app system. An instance of the DB is offloaded onto the handheld for offline use. Changes to the DB are reflected in handhelds after an update cycle, as the model does not presume that network access will be continuous [8]. The web-based administrator interface allows the medical domain expert(s) to upload, edit and assure correctness of data stored in the DB. Through the CDS app system, end users have either a website or handheld view of the medical data stored in the DB as a source for their decision making. app: Mobile application; CDS: Clinical decision support.

**Figure 3.**
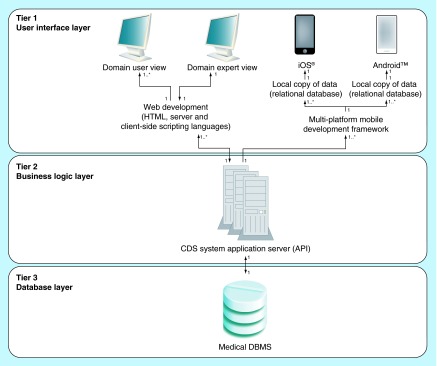
**The Bridging Opportunities Work-frame software architecture components organized in a three-tier model.** The architect framework's goal is to enable mobile tools to deliver decision support at point-of-care in order to improve the use of medications or the selection of treatments. API: Application Programming Interface; CDS: Clinical decision support; DBMS: Database management system; HTML: Hypertext markup language.

**Figure 4.**
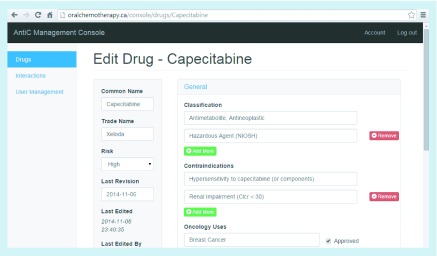
**The Bridging Opportunities Work-frame model calls for an administrator interface.** This figure shows the administrator console for use by the healthcare provider in our test case. The web-based interface allows real-time updates for adding and making changes to stored data.

**Figure 5.**
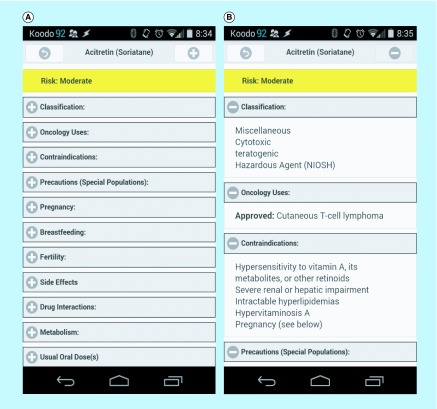
**The Bridging Opportunities Work-frame model is designed to create clinical decision support mobile applications.** This figure demonstrates the clinical interface of our test-case app on an Android platform. Two views are shown based on our test case: **(A)** high-level headings grouping available data that are consistent across categories; **(B)** subcategories can be opened independently to drill down to additional information. app: Mobile application.

The efficient translation of evidence-based science to point-of-care is a well-known problem [1–3]. Uptake can be hampered by a lack of delivery systems or by the absence of required policy changes [3–5]. According to a recent scoping review (a review category that outlines existing research), the use of handheld computers is on the rise in clinical settings where they are used by healthcare professionals for patient documentation, patient data access and general information lookup [6]. However, there are few examples of their use in clinical decision support (CDS) with the aim of translating evidence-based data to point-of-care [6,7]. Handhelds may be most effective when used in time-constrained settings, but effectiveness and outcomes research is generally lacking [6,7]. Most documented decision support is not specific to a particular workflow and simply concerns the use of digital versions of generally available drug information [7,8]. However, when based on locally developed guidelines, decision support is accessed frequently and has a measurable impact [8,9]. For example, based on this premise, handhelds have been used in an outpatient setting to improve treatment decisions for the prescription of nonsteroidal anti-inflammatory drugs [8]. And, in a critical care setting, tailored decision support led to reduction in the number of antibiotic prescriptions, as well as the overall length of a patient's hospital stay [9]. A mobile application (app) on a smartphone providing evidence-based recommendations and medication monographs was found to be effective for guiding antidepressant drug selection [10]. Improving access to relevant context-specific data for use by healthcare providers is predicted to have a direct effect [11,12].

At present the most popular handheld platforms, based on overall market penetration as well as current use in clinical settings, are Android™ and iOS^®^ devices [13]. Driven by the ubiquity of these mobile technologies and an increasingly health-aware population, there has been a recent surge of mobile health application software development, also known as mHealth [1,14–15]. Decision support presents an innovative possibility in the use of mHealth [16]. Mobile applications, or apps, are, in principle, well positioned to provide CDS to healthcare providers, as they could help to address real-time deficiencies in medical knowledge, or to translate new evidence-based knowledge within the context of a patient's visit [1,2]. Indeed, medical professionals indicate that they would like to use more CDS apps, but note that there is currently a deficiency of high-quality decision support apps available [13].

Many hurdles exist when trying to implement mobile health applications with an aim to provide on-the-spot decision support. CDS systems are complex [4–5,17]. The creation of medical CDS systems, including CDS apps, can be hindered by a lack of expert input, or a lack of technology implementation know-how, or both [3–5,17]. Additional obstacles lie in project organization, expertise, data interpretation, content creation, content revision and appropriate visualization or presentation [1–2,15]. The design and implementation of decision support tools in an mHealth context is sufficiently wide ranging that none of the participants can fully understand all intricacies of the project as a whole, necessitating multidisciplinary teams [18]. Stakeholders’ core competencies and capabilities should align with specific concerns around knowledge management [19]; a clear definition of roles and responsibilities of different domain experts in the development process therefore becomes a prerequisite. Furthermore, upon completion, all CDS apps require ongoing involvement of a medical expert [20].

In dealing with complexity in large-scale eHealth projects, enterprise architecture (EA) approaches have been successfully applied [21]. In eHealth, EA looks at both information technology (IT) architecture and business processes to create the change required to achieve a particular strategy [22]. In other words, EA combines an IT architecture framework with an organizational chart to help implement new systems. User acceptance of new systems can be increased by involving end users in the development process [23], and user empowerment in quality assurance has been brought forward as a critical tool for end-user sign-off [24]. Furthermore, empowerment fosters commitment and increases awareness of the usability and usefulness of a software solution [19,23–24]. Additionally, as an IT architecture framework, three-tier client/server architectures have known advantages for maintenance, reuse and flexibility in clinical information systems settings [25].

Based on this, and in order to address the obstacles to CDS development, we hypothesized that CDS app development would benefit from a small-scale EA model to address project complexity, stakeholder roles and responsibilities, end-user acceptance and ongoing involvement of an expert healthcare provider. Our aim was to create and test a generic, re-usable model that would make it possible for healthcare providers, who are domain experts, to guide the development of CDS apps, as they create a system to upload and edit evidence-based data to be integrated into decision-making processes. To this end we created a Bridging Opportunities Work-frame (BOW) model.

The test case for the application of our BOW model was provided through a project to improve and promote the community pharmacist's role in oral chemotherapy medication management. Taking chemotherapy in tablet form has obvious advantages for the patient, who can avoid disruptive and time-consuming hospital visits to receive chemotherapy. Furthermore, there appears to be an overall cost saving due to the lack of hospitalization to receive the therapy [26]. However, this leads to higher information access requirements for pharmacists [27], and non oncology-trained pharmacists have to deal with the use of chemotherapy outside of a hospital environment. There is a perceived increase in the risk of patients taking their medications incorrectly [26,27]. We predict that handheld access to context-specific information on the use of these generally toxic medications would be pertinent to community pharmacists who are not trained in the safe handling and dispensing of cytotoxic medications, and thus could help prevent errors and improve patient outcomes. Using a BOW, from development through testing to user acceptance, a CDS app containing curated and up-to-date Canadian data on oral chemotherapeutics was created for use within the Canadian community pharmacist's normal workflow. While filling prescriptions, pharmacists can, without difficulty, check for issues such as prescription errors, drug–drug interactions, treatments for comorbidities and medication side effects. This study developed and tested the BOW methodology to formalize CDS app development, where a gap in the workflow has been identified by a healthcare provider and evidence-based knowledge is available to address this.

## Methodology

### BOW model

We built a BOW model consisting of two parts, one covering the governance of organizational changes and one covering the use of IT:

We utilized Lewin's Change Management model [28,29], to alter roles and responsibilities in the organization of the development team by establishing a central leadership role for the healthcare provider in the CDS app creation process, and shifting the software development expertise on the team to a leading from below role [30];
For the information architecture framework, we applied a three-tier client/server architecture as described by Eckerson [31], and others [25], to the storage, organization and presentation of evidence-based medical data underlying CDS apps.

### Case study

43 monographs, representing the current state of use of oral chemotherapeutics in Canada, were created and curated by an oncology-trained pharmacist. These included the generic and trade names of medications, their classification, their indications and approved oncology uses, contraindications, precautions both for general and special populations, pregnancy and breastfeeding precautions, effects on fertility, side effects, drug interactions, metabolism, usual oral dosages, dose adjustments, excretion, unit dose availability, paths of administration and monitoring concerns. Risk levels were assigned to each drug to further warn of drug–drug interactions, therapeutic duplications and dosages that are unsuitable for special populations. This was combined with data on drug interactions due to CYP metabolism and p-glycoprotein efflux pump activity. Protocols and dose adjustment tables dealing with either myelosuppression or decreased liver function are also included. In keeping with the outline provided by a BOW, this work was done by the medical domain expert who was, in this case, an oncology-trained pharmacist.

### BOW model application

We tested the applicability of the BOW model by developing a CDS app to assist pharmacists in filling anticancer medication prescriptions:

A three-tier application was created through the development expertise using the following steps:
  – A code repository was created on GitHub to allow for version control [32];
  – An entity-relationship (ER) model was shaped based on the data provided by the pharmacist;
  – A relational database (DB), MySQL™ 5.5.32 for Debian Linux, was put in place based on a schema derived from the ER diagram;
  – A Hypertext Preprocessor (PHP) server side Application Programming Interface (API) was developed to control access to the DB and to provide the business logic layer for client requests;
  – A PHP microframework based on the Silex framework was used for the web application server side of the user and administrative views;
  – The DB, API and web application server were run on Ubuntu Linux 13.10 on Amazon Web Services™ Elastic Compute Cloud (EC2);
  – Hypertext Markup Language (HTML) 5, JavaScript^®^ and Cascading Style Sheets were used to develop the web application client side, using the Twitter Bootstrap^®^ framework to facilitate user interface (UI) design;
  – Apache Cordova™ PhoneGap 2.3.0, a mobile development framework in JavaScript, was used for cross-platform functionality spanning iOS and Android. It was used for both the graphical UI (GUI) design for the handhelds and access to data through the API;
  – Local data storage was handled through Cordova's DB implementation to store data in Android's SQLite 3.7.11 for use on Android 4.1–4.4, and a SQLite 3.7.13 for use on iOS 7;
Direction of development, testing and signing off on interfaces and functionalities was performed by the pharmacist;
Curation of data was performed by the pharmacist;
Data integrity and security were performed by the development team;
Installation Qualification and Operational Qualification were performed by the development team;
Alpha-testing (simulated operation testing by a potential user) was performed by the pharmacist;
Software updates, fixes and speed of response improvements were handled by the development team;
Validation and acceptance of updates were performed by the pharmacist.

Data curation and Performance Qualification are ongoing activities performed by the medical domain expert, in this case the oncology-trained pharmacist. Design Qualification was provided by using a BOW model. For the web-enabled interfaces, JavaScript was used client side to enhance the user experience and speed of the application.

## Results

### Creation of a BOW model

We created the BOW model for CDS app development. We built the view model based on Lewin's Change Management model [28,29], to organize stakeholder's roles and responsibilities as shown in Figure 1, and worked through their interaction with the IT components as shown in Figure 2. For the IT architecture framework, the BOW model applied three-tier architecture as shown in Figure 3. The purpose of the organizational workflow and IT architecture was to create a system to convey complex information in an instant, appropriately organized and tailored for use in a time-constrained medical environment [19,24,33].

In the BOW model developed in this study we strictly defined the roles of stakeholders and overall software architecture and divided them into their functional parts (Figures 1–3). The healthcare provider, as a medical domain expert, was the project lead and in control of all data organization, visualization, presentation, acceptance and dissemination. The software development team, classified as application development expertise in the BOW model, worked on component development to support the aims of the healthcare provider and parsed the data provided (by the medical domain expert) to extract its semantics, in order to create a DB schema. Additionally, the application development expertise provided an access point for the medical domain expert to autonomously edit and update the CDS data (Figure 4). The medical domain expert is similarly a medical domain user; consequently, quality verification of UIs and the cogency of the CDS app were achieved by iterative testing throughout the development.

The central axis of the software framework was the CDS app system's API, as it connected a relational Database Management System (DBMS) to a web application, providing both user and administrator interfaces. The DBMS housed the data, the API provided the business logic and powered the interfaces for adding, editing and displaying data. Together, these components formed a three-tier structure (Figure 3) [25,31].

### BOW model application

A case study was used to test both the validity of the architect framework and the project management assumptions behind the BOW model. As per the BOW model, the project was directed by a medical domain expert. An app was developed to provide decision support to community pharmacists in safely filling anticancer medication prescriptions.

Comprehensive, curated and relevant data on 43 oral chemotherapeutic medications available in Canada were implemented for use on handhelds. The oncology pharmacist provided the evidence-based data, which was well formed, making parsing of the data easier [34] because it was rule based and had a high degree of data organization (e.g., medication monographs, dosing adjustment rules, cautions, interactions, etc.). Exploiting this particular property, ERs, classes and logic rules were derived [34]. The software development team created the components of the BOW models’ three-tier architecture.

The first tier's GUIs consist of views from both workstations as well as handheld Android and iOS devices. For the second tier an API was created, which, through a web server, processed the requests to the data from the various GUIs. The third tier housed the DBMS allowing for the storage of, and transactions to, the oral chemotherapeutic data.

The software has specific forms and views for both user and administrative roles. Web interfaces were made available through a website for laptop and desktop access [35]. On handhelds, view-only data are made available on devices based on either Android (smartphone, tablet) or iOS (iPhone^®^, iPod^®^, iPad^®^) platforms. Figure 5 shows the dedicated CDS app interface for the oral chemotherapeutic drug acitretin on an Android platform. A single relational DBMS provides the main content for every instance of the tool set. A unique instance of the DB is downloaded on handhelds to provide a local data copy for offline data use; this copy of the content is stored directly on the smartphone or tablet and assures access to data in the absence of a Wi-Fi or mobile signal. Changes to the DBMS are reflected simultaneously on a website and, in the presence of a mobile or network connection, as an available update on their mobile counterparts. Updates are pushed with a user prompt when a connection becomes available.

## Discussion

We developed and applied a BOW to app development (Figure 5), providing a reusable model for future development of healthcare apps. In our test case we involved an oncology-trained pharmacist as the healthcare provider, to create a system to make relevant data on oral chemotherapeutics available on handhelds for use by Canadian community pharmacists. In keeping with the amount of display available on handhelds and not to overwhelm the user with extraneous data, our test case was focused on pharmacists’ use within a regional (Canadian) context. An administrative interface was built to allow the healthcare provider to autonomously edit and update the source data (Figure 4). The app system links data from medication monographs to interactions, dose adjustments and protocols. Once tested and validated by a large group of end users (in this case, hospital and community pharmacists), this app could be presented on handhelds, and oral chemotherapeutic knowledge could be incorporated in the day-to-day workflow of community pharmacists (Figure 5) [36].

In order to increase stakeholder engagement, we altered the software development process [28–29,37]. In addition to an architect framework, BOW is a project management model where the healthcare provider becomes the leader of the software development project rather than a client to it [30]. In doing so, our BOW addresses common issues affecting the success of electronic healthcare system development such as sensitivity to the medical context, development issues, equipment selection, uptake and maintenance [4–5,38]. Additionally, it ensures known facilitators such as ease of use, reliability and integration in the day-to-day workflow [38,39].

In our test case, the application of the generic BOW model made it possible for an oncology-trained pharmacist to guide the development of a CDS app, and create a system to upload and edit evidence-based data to be integrated into pharmacy decision-making processes regarding the dispensing of anticancer drugs. Furthermore, as a user in the healthcare field directed the development of the software's abilities, we expect this to benefit uptake and dissemination of our app in Canadian pharmacy settings, once tested and validated by a larger team of end users [19,23–24]. Based on the BOW model's implementation in our oral chemotherapy app development case study, we believe this mHealth approach to be generalizable across similar situations, where specific medical frontline workflows would benefit from tailored decision support integration.

### Limitations

The BOW model's architecture framework is not completely independent of the corresponding implementation, as currently app development has few choices in development tools. In addition, medical data are inherently complex and as research continues, or fine-tuning based on clinical outcomes becomes available, new guidance will emerge [3–5]; hence, a CDS app can never truly be completed without becoming obsolete [2]. This necessitates ongoing updates to any medical app, but even more so for CDS apps that are used to help healthcare providers determine an appropriate medical course of action in real-time settings [2]. Thus, a healthcare provider's commitment is necessary to keep a CDS app system up-to-date [19,23–24]. The development of an app for healthcare, even with the inclusion of a healthcare provider at the development stage, does not preclude the need for regulatory approval from country-specific agencies before the app can be used in a healthcare setting. A generic model for app development does not automatically guarantee regulatory approval, and the quality of input data and quality of testing and validation are crucial to app success. Finally, CDS apps developed through a BOW model will, like most apps, need some modifications to continue functioning when major operating system revisions occur on handheld platforms.

## Conclusion

We developed a generic model to build CDS apps. In this instance we used a test case aimed to improve the safe handling, dispensing and use of oral chemotherapeutics outside of the hospital setting. Through a secure web-based administrator interface, oncology-trained pharmacists can curate the data as new oral chemotherapeutic agents or guidance on their use becomes available. Once tested and validated for accuracy of content, using this CDS app, community pharmacists could actively apply current evidence-based knowledge on safety and standards in the use of oral chemotherapeutics.

The CDS app and website are linked to the same data source; however, handhelds can be used as a standalone whenever network or mobile connections are not available (e.g., rural settings) [40]. If there are changes or updates to the source data during this time, pharmacists will be alerted to an automatic update as soon as a connection becomes available again. This brings knowledge to the bedside at the touch of a button.

The test CDS app was developed on the re-usable BOW model. We envision that the generic template provided by our BOW model could be applied to other healthcare scenarios where having instant access to complex and dynamic evidence-based knowledge would be vital for improving patient outcomes. Moreover, the genesis of a separate software package based on the model developed here could enable non-technical users to create fully functioning mobile CDS apps by themselves, akin to the way in which blogs allowed laypersons web publishing [41]. We believe that the model presented here can help spur the growth of evidence-based medicine adoption by lowering the barrier to entry for healthcare providers looking to use mHealth to address known knowledge gaps [40,42]. More case studies are needed to assess the feasibility of disruptive innovation based on the BOW model [43].

## Future perspective

Although certain therapies have been proven to be efficacious, they are not being used in clinical settings. CDS systems can help to move local recommendations and guidelines to healthcare providers so they can integrate them in their decision-making processes when providing care for patients [44]. The aim is to do this in a manner that helps to improve both the quality and safety of the care delivered [45]. CDS development will be increasingly initiated by healthcare providers in a ‘user pull’ model [45], as opposed to a ‘vendor push’ of prepackaged and ill-fitting solutions. Stakeholder engagement is essential, and significant preprocessing of medical data will become a prerequisite. Indeed, this role is already predicted for pharmacists in the translation of pharmacogenomics [46]. Current approaches such as support vector machines and decision trees are already deployed [39,47]; in addition, CDS data need to be locally applicable and devoid of clutter. Further, any CDS apps developed using the BOW model need to undergo robust curation of the input data and extensive field testing before being submitted to country-specific regulatory agencies for approval to use in healthcare settings. In mHealth, CDS constitutes a higher level objective of medical data usage with significant potential impact. We predict that standardized medical app development involving end users, like in the model presented here, will grow rapidly in the near future.

**Executive summary**

**Background**

Clinical decision support (CDS) mobile applications (apps) may show the greatest promise when created by and for frontline clinicians, as they identify and address shortcomings in their current workflow.
This study aimed to create and test a generic model that makes it possible for healthcare providers, who are domain experts, to guide the development of CDS apps, as they create a system to upload and edit evidence-based data to be integrated into decision-making processes.

**Methodology**

Organizational Change Management was combined with a three-tier information technology architecture, separating medical data from engineering concerns, to enable healthcare providers to lead CDS app development.
Testing the validity of the model, a specific CDS app was developed to make up-to-date information on oral chemotherapy agents available to Canadian pharmacists.

**Results**

The Bridging Opportunities Work-frame (BOW) model was created.
The BOW model strictly defines the roles of stakeholders and overall software architecture in order to stimulate development and implementation of CDS apps.
Using a BOW, a CDS app was developed based on Canadian oral chemotherapy use.

**Discussion**

Healthcare provider generated and curated data, presented on pharmacists’ cue using handhelds has the potential to translate into application of evidence-based medicine in emerging medication use or novel treatment regimens.
The BOW model increases stakeholder engagement by altering the software development process so the healthcare provider becomes the leader of the software development project rather than a client to it.
The use of the BOW addresses common issues affecting the implementation success of electronic healthcare systems.

**Conclusion**

The BOW model presents a re-usable, potentially disruptive innovation approach to CDS app development.
Globally applicable, the BOW model allows regional healthcare providers to direct the process of placing decision support information onto handhelds.

**Future perspective**

CDS development is a high value activity in mobile medical app development, and subsequently will experience rapid growth in the near future.
Rather than using a ‘one size fits all’ commercial off the shelf solution, healthcare providers will actively be involved in the development of CDS apps for their own domains and locales.
